# Preserved circadian variation in cortisol and androgens during a ski traverse of Antarctica in summer

**DOI:** 10.1038/s41598-025-01165-1

**Published:** 2025-05-22

**Authors:** Robert M. Gifford, Natalie Taylor, Jack Kendall, John Hattersley, Natalie Z. M. Homer, David R. Woods, Christopher Imray, Adrian J. Wilson

**Affiliations:** 1 Academic Department of Military General Practice, Royal Centre for Defence Medicine, Edgbaston, UK; 2 Carnegie School of Sport, Leeds Beckett University, Leeds, UK; 3 Human Metabolic Research Unit, University Hospitals Coventry and Warwickshire NHS Trust, Coventry, UK; 4 Mass Spectrometry Core, Centre for Cardiovascular Science, University of Edinburgh, Edinburgh, UK; 5 Academic Department of Military Medicine, Royal Centre for Defence Medicine, Edgbaston, UK; 6 Department of Vascular and Renal Transplant Surgery, University Hospitals Coventry and Warwickshire NHS Trust, Coventry, UK; 7 Department of Research and Development, University Hospitals Coventry and Warwickshire NHS Trust, Coventry, UK; 8 Department of Physics, University of Warwick, Coventry, UK; 9 Centre for Cardiovascular Science, Queen’s Medical Research Institute, University of Edinburgh, Edinburgh, UK 47 Little France Crescent, EH16 4TJ

**Keywords:** Homeostasis, Thyroid gland, Reproductive biology

## Abstract

Antarctic expeditions present extreme physiological challenges due to cold temperatures, high physical exertion, and 24-hour daylight. This observational study evaluated endocrine adaptation in nine participants (six men, three women) during a 47-day, 1,000 km unassisted ski traverse. Detailed salivary sampling was conducted before, during and after the expedition, corroborated by blood and hair sampling before and after the expedition. Cortisol, testosterone, and androstenedione were measured using mass spectrometry, and thyroid hormones via immunoassay. Diurnal cortisol, androstenedione and testosterone variation was preserved, while the morning cortisol increased during the expedition, suggesting that exercise demands overshadow the effects of continuous daylight in controlling hypothalamic-pituitary-adrenal and gonadal axis function. Morning testosterone decreased during the expedition, with a greater effect seen among men. No significant changes were seen in blood or hair steroid hormones. Gonadotropins in women indicated central suppression pre-expedition, normalizing post-expedition. Thyroid-stimulating hormone levels increased post-expedition without significant changes in free T3 or T4, consistent with mild polar T3 syndrome. These findings highlight the adaptability of hypothalamic-pituitary function to combined stressors of exercise, energy deficit, and cold. This is the first study to capture in situ endocrine responses during an Antarctic traverse, advancing our understanding of human adaptation in extreme environments.

**Supplementary Information:**

The online version contains supplementary material available at 10.1038/s41598-025-01165-1.

## Introduction

Polar regions present one of the planet’s most austere environments for exercise, exposing individuals to high winds, low temperatures and isolation. Unassisted ski touring involves high levels of physical activity (estimated to be burning 6,000–8,000 kcal/day^1–3^) in 24-hour daylight, with a day-to-day schedule which remains constant over several weeks. An energy deficit is normally observed^2–4^ but not invariably so^1^. In combination, these stressors place complex demands on physiology, mediated through changes in the function of glands under the control of the anterior pituitary, in particular the adrenals, gonads and thyroid^5^.

Cortisol is released by activation of the hypothalamic-pituitary-adrenal (HPA) axis which upregulates energy substrate to meet demand. Cortisol concentrations normally vary according to the circadian rhythm which is critically regulated by the light-dark cycle^6^, increasing at dawn and decreasing through dusk into the night time. Exposure to light in the early morning increases the cortisol response to awakening^7^, whereas light exposure throughout nighttime reduces the morning rise in cortisol and flattens diurnal variation^8^. Androgens, such as testosterone, promote the generation and maintenance of skeletal muscle^9^. Concentrations of cortisol and androgens vary according to external factors like time of day, cold, exercise intensity and duration, and individual factors including biological sex, energy balance and the sufficiency of sleep. Steroid concentrations can be measured from blood and saliva samples, but average steroid concentrations can be measured over longer durations from hair. Since hair grows at a constant rate and steroids remain stable in hair for several months, hair steroids can provide a retrospective average concentration over long durations. This is complementary to diurnal steroid variation from point measures and helps provide a more comprehensive profile of steroid production. Hair samples also have the advantage in adverse environments of not requiring specialist collection devices, pre-processing or storage.

The hypothalamic-pituitary-thyroid (HPT) axis adapts to sickness and prolonged fasting^10^, exercise and negative energy balance^11^ and polar environments^12^. The extreme cold of polar environments induces a HPT axis pattern known as “polar T3 syndrome”^13^. In prolonged exposure to subzero temperature, concentrations of triiodothyronine (T3, the active thyroid hormone) fall, and thyroxine (T4, the inactive, more stable thyroid hormone) and thyroid stimulating hormone (TSH) are normal or slightly elevated^13^. Normally the HPT axis helps regulate the hypothalamic-pituitary-gonad (HPG) axis via common signalling pathways. Disruption in thyroid function leading lead hypothalamic amenorrhoea, impaired spermatogenesis and reduced androgen biosynthesis^14,15^.

Previous studies have documented hormonal changes during exposure to polar environments. In 9 men living in Antarctica for a year, monthly blood samples demonstrated higher summer testosterone levels, which appeared to be driven by hypothalamic and pituitary activity^16^. In 6 women undertaking a 61-day Antarctic ski traverse, no change in testosterone was seen 1 week afterwards and gonadotroph response was preserved, despite increased average cortisol exposure measured in hair^3^. Nineteen men demonstrated increased testosterone levels after a less arduous 42-day Antarctic ski expedition^1^. Significant sex differences are expected in hormonal adaptation to exercise, however few studies have compared male and female responses to exercise in polar environments. One study assessed sex differences in testosterone, cortisol and thyroid hormones in 10 participants (3 female) undertaking sailing and camping in Antarctica. Morning cortisol levels were higher among women during camping, while testosterone levels (higher among men) did not differ significantly. Diurnal levels of cortisol and testosterone were blunted while exposed to 24-h daylight^17^, however the limited sampling frequency and sedentary nature of this expedition limited its applicability for exercise adaptation in this setting. Such studies are frequently limited by the practical challenges of measurement ‘on the ice’, and most have resorted to pre- and post- expedition measures and cannot determine the temporal nature of adaptation. These studies have also been limited by the use of enzyme immunoassays, which lack analytical accuracy, precision, and range compared to measurement using - mass spectrometry (particularly at the low testosterone levels found in women)^18^.

We undertook an exploratory, observational study describing endocrine responses during an Antarctic ski traverse, using ‘on-ice’ measurements to measure cortisol and androgens in saliva with gold-standard tandem mass spectrometry methodology. This study assessed circadian changes in cortisol and androgens during a 1,000 km unassisted Antarctic ski traverse undertaken by 6 men and 3 women. We aimed to determine circadian changes in cortisol and androgens before, during and after the expedition, corroborated by measures of these steroids in hair and blood, and by thyroid hormones assessing the degree of physiological adaptation to cold. We hypothesised that cortisol would increase and androgens decrease during the expedition, but the diurnal variation in both would flatten with 24-hour daylight^19,20^. We also hypothesised that the cortisol rise would be greater in women than men, greater flattening of diurnal variation would be seen among men, and from the start of the expedition, androgens would decrease relative to cortisol to a greater extent in women compared with men.

## Method

The INterdisciplinary South Pole Innovation and Research Expedition 22 (INSPIRE 22) was a 47-day ski traverse from the Antarctic Ronne Ice Shelf to the South Pole, 2022–2023 undertaken by 9 participants (6 male). The mean age of participants was 40.0 (SD 10.3) years at the start of the expedition (male participants ranged 28 to 61, female participants ranged 37 to 41). Of three women, one used a long-acting progesterone-eluting intrauterine device (Jaydess^®^), one used a progesterone-only pill continuously, and one used no hormonal contraceptive, throughout the study. Inclusion criteria for the scientific study were participation in the expedition and self-declared as physically fit to do so. Exclusion criteria were pregnancy or usage of medication requiring refrigeration. The expedition comprised a fixed daily routine of hauling sledges for 7 h, covering a total 911 km and 2,800 m ascent. The expedition’s physical demands and physical changes are described in Wilson et al.^21^.

Ethical approval was obtained from the Ministry of Defence Research Ethics Committee (2125/MODREC/22). The study was conducted in accordance with the Declaration of Helsinki. All 9 expedition members volunteered to take part in the study and provided written informed consent before any measurements were made.

### Study protocol

Cortisol and androgen profiles are highly variable throughout the day, so it is important they are sampled at precise time points^22^. Four to 6 weeks before the expedition (median 30 days, denoted ‘pre-30 days’), a detailed salivary ‘day curve’ was conducted comprising 9 samples over 24 h (06:15 on waking, 06:30, 06:45, 07:00, 07:15, 11:00, 15:00, 20:00 and 22:00). Subsequent saliva samples were taken at 06:00, 06:15, 06:30, 15:00 and 20:00. This 5-point profile was conducted 14 days before the start of the expedition (Pre − 14 days), on the first day and after 15 days and 30 days of skiing (Ex days 1, 15 and 30, respectively), and 1 and 6 days following the completion of the expedition (Post + 1 day and Post + 6 days). Ten to 16 days (median 13 days) after the expedition, the detailed 9-sample profile was repeated (Post + 13 days).

Saliva was sampled by placing a nylon swab in the mouth for 30 s (Salivette ^®^; Sarstedt, Nümbrecht, Germany). Samples were stored at 5 ℃ or lower for up to 7 days prior to being frozen at − 20 ℃ until analysis.

Hair was sampled on 3 occasions: 2 weeks prior to the expedition, at a resupply point on day 21 of the expedition, and 4 weeks thereafter. A 5 mm diameter bundle of hair was cut from the posterior vertex region of the head, as close as possible to the scalp. Hair was stored in aluminium foil at ambient temperature and analysed for cortisol and testosterone by liquid chromatography tandem mass spectrometry (LC-MS/MS) by Dresden Lab Service GmbH (Dresden, Germany). Samples were cut into 1 cm segments, assuming an average growth rate of 1 cm per month. The two most proximal segments were assayed from the first two hair samples, and the most proximal segment from the third, providing five months of average exposure (3 months before and 2 months during the expedition).

An early morning blood sample was taken after an 8-hour fast, at Pre − 30 days, Pre − 14 days, Post + 6 days and Post + 13 days. Blood was sampled in ethylenediaminetetraacetic acid and serum-separating gel tubes (Monovette^®^, Sarstedt, Nümbrecht, Germany), transported in dry ice and stored at − 80 ℃ until analysis.

Targeted liquid chromatography/ tandem mass spectrometry (LC-MS/MS) was used to measure the concentrations of cortisol, androstenedione and testosterone in saliva and plasma. The method for saliva steroid measurement has previously been reported in Gregory et al.^23^ and the method for plasma steroid measurement has previously been reported in Denham et al.^24^ In brief, saliva or plasma (200 uL) were enriched with isotopically labelled steroids as internal standards, extracted using supported liquid extraction alongside calibration curves that covered expected physiological concentration ranges of glucocorticoids and androgens in plasma and saliva. Extracts were separated on a Kinetex C18 (150 × 2.1 mm; 2.6 μm) column using water and methanol with 0.05 mM ammonium fluoride. Eluent was subject to electrospray ionisation and polarity switching with tandem mass spectrometry in multiple reaction mode. Amounts of steroids in plasma and serum were calculated using linear regression of peak areas of the steroids/internal standards.

Serum free T3, free T4, TSH, sex hormone binding globulin (SHBG), luteinising hormone (LH), and follicle stimulating hormone (FSH) were measured in serum using Abbott Architect^®^ analyser according to the manufacturer’s instructions. Free testosterone was derived using an online calculator^25^.

### Statistical analysis

The trapezoidal rule was used to calculate cortisol area under the curve, for the earliest 45 min waking cortisol during each sampling period (awAUC) and for all samples in the 9-point day curve at Pre − 35 days and Post + 13 days (dayAUC). Diurnal slope was calculated as the difference between the maximum cortisol measured over the first 45 min (06:15 to 07:00) minus the cortisol measure at 20:00 on the same day, scaled by the time difference in hours^26^. Androstenedione: cortisol ratios were calculated from the highest measured value and the value at 20:00, on each sampling day.

Only awAUC and morning cortisol values were included in analysis for the Post + 1 day visit due to the missing 20:00 sample. Other missing values were replaced by linear interpolation (one 06:30 sample in Pre – 30 days). In a small number of cases the concentration of the analyte was too small to analyse and a value of the lower limit of detection was used (e.g. one female 20:00 sample for testosterone taken at Pre − 14 days).

Due to the sample size, normality of distributions could not be assessed, so, as is conventional, non-parametric statistics were used. We conducted a multi-way ANOVA on ranks of data using the aligned rank transform (ART) of Wobbrock et al.^27^ ART was chosen over univariate statistics, since it has the advantage of utilising the full variance in the data set and allows analysis of off-axis differences resulting from interaction between variables.

Spearman’s rank order correlation was conducted to compare salivary and serum testosterone and androstenedione.

Whilst the use of small sample non-parametric statistics provides a method of quantitively analysing the data from our study, the small number of subjects, particularly the small number of women, mean that a cautious approach to interpretation of the results of the analysis has been adopted throughout.

Data were processed and plotted using Python (version 3.9) or GraphPad Prism for Mac OS (version 10; graphpad.com) and the statistical tests performed using R (version 4.3.1)^28^.

## Results

Overall compliance with the study protocol was high, however no 20:00 saliva time point was taken on Post + 1 day due to an unforeseen conflict with other parts of the protocol.

**Fig. 1:**
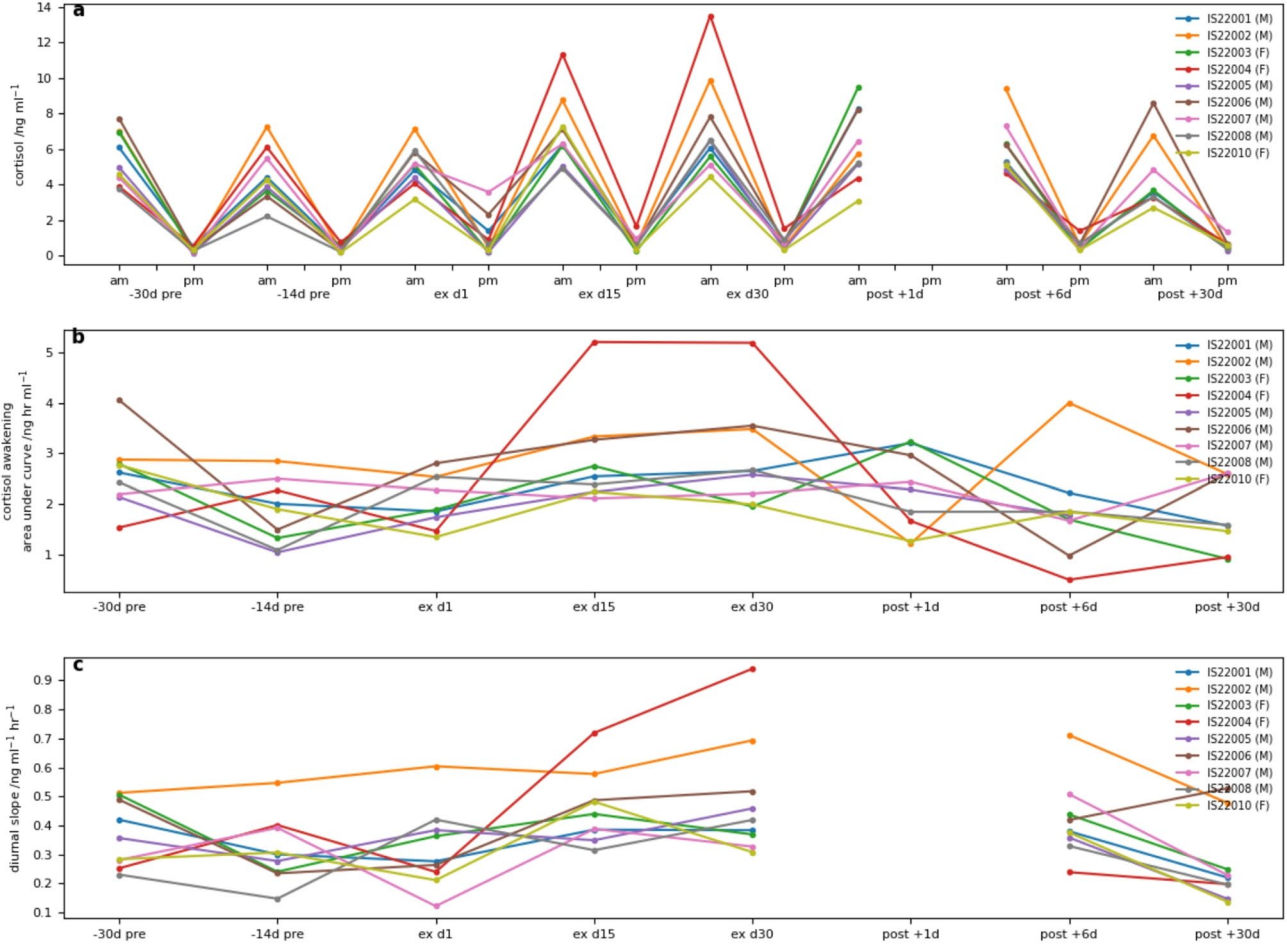
Salivary cortisol concentrations before during and after the expedition. (**a**) Peak morning and evening cortisol concentrations. (**b**) Cortisol awakening area under curve calculated by the trapezoidal rule; (**c**) Diurnal slope for cortisol. Individualised data. F: Female participant; M: Male participant. For statistical analyses, see main body.

Salivary steroid hormone concentrations are shown in Table 1. Pre-expedition morning salivary cortisol levels demonstrated consistent, significant diurnal variation throughout the study. Morning cortisol, awAUC and diurnal slope increased during the expedition and then normalised to baseline levels within 13 days. No sex difference was observed in cortisol concentration, awAUC or diurnal slope, however a significant sex × time interaction was demonstrated across all cortisol samples, suggesting a greater increase among women than men during the expedition. Figure 1 shows individual data. A marked rise in morning cortisol was observed in participant 4, and participant 3 in post Ex + 1d (both female). A high evening salivary cortisol was observed in 3 out of the 6 male participants at Ex − 14d, possibly reflecting greater anticipatory stress.

**Table 1:** Saliva steroid hormone concentrations given in Ng/dL.

		Pre − 30 days	Pre − 14 days	Ex day 1	Ex day 15	Ex day 30	Post + 1 day	Post + 6 days	Post + 13 days	*p* (time)	*p* (time of day)	*p* (time × time of day)	*p* (sex)	*p* (sex × time)
Cortisol														
AM	Male	5.66 ± 1.55	4.43 ± 1.76	5.54 ± 0.977	6.39 ± 1.43	6.98 ± 1.67	6.51 ± 1.42	6.39 ± 1.72	4.9 ± 2.37	0.0001	0.0001	0.6	0.59	0.034
	Female	5.15 ± 1.61	4.68 ± 1.28	4.12 ± 0.974	8.28 ± 2.71	7.85 ± 4.94	5.64 ± 3.37	5.34 ± 0.857	3.23 ± 0.489	F (6, 91) = 5.26	F (1, 91) = 379.16	F (1, 91) = 0.27	F (1, 7) = 0.319	F (6, 91) = 2.39
PM	Male	0.302 ± 0.137	0.323 ± 0.067	1.45 ± 1.3	0.679 ± 0.145	0.666 ± 0.205		0.595 ± 0.132	0.61 ± 0.386					
	Female	0.378 ± 0.152	0.503 ± 0.289	0.479 ± 0.371	0.765 ± 0.767	0.812 ± 0.635		0.704 ± 0.607	0.557 ± 0.147					
awAUC														
Male		2.72 ± 0.71	1.83 ± 0.747	2.29 ± 0.419	2.65 ± 0.527	2.86 ± 0.538	2.33 ± 0.727	2.08 ± 1.02	1.97 ± 0.735	0.013			0.29	0.61
Female		2.36 ± 0.719	1.83 ± 0.476	1.57 ± 0.284	3.4 ± 1.59	3.04 ± 1.86	2.06 ± 1.04	1.35 ± 0.737	1.11 ± 0.307	F (7, 49) = 2.901			F (1, 7) = 1.31	F (7, 49) = 0.77
Diurnal slope														
Male		-0.382 ± -0.113	-0.316 ± 0.138	-0.345 ± 0.164	-0.417 ± 0.097	-0.467 ± 0.128		-0.451 ± 0.142	-0.3 ± 0.161	0.005			0.79	0.41
Female		-0.347 ± 0.138	-0.316 ± 0.08	-0.272 ± 0.08	-0.547 ± 0.15	-0.539 ± 0.349		-0.351 ± 0.102	-0.195 ± 0.056	F (6, 42) = 3.692			F (1, 7) = 0.08	F (6, 42) = 1.05
Testosterone														
AM	Male	0.087 ± 0.021	0.073 ± 0.019	0.098 ± 0.032	0.08 ± 0.021	0.082 ± 0.041	0.100 ± 0.090	0.069 ± 0.009	0.092 ± 0.011	0.016	< 0.0001	< 0.0001	0.0001	0.014
	Female	0.01 ± 0.003	0.013 ± 0.012	0.01 ± 0.001	0.019 ± 0.022	0.012 ± 0.003	0.012 ± 0.005	0.014 ± 0.007	0.01 ± 0.001	F (6, 91) = 2.77	F (1, 91) = 155.66	F (1, 91) = 79.57	F (1, 7) = 38.915	F (6, 91) = 2.83
PM	Male	0.042 ± 0.007	0.032 ± 0.008	0.043 ± 0.01	0.033 ± 0.007	0.031 ± 0.011		0.037 ± 0.02	0.042 ± 0.012					
	Female	0.005 ± 0.001	0.004 ± 0.004	0.003 ± 0.001	0.005 ± 0.001	0.029 ± 0.039		0.005 ± 0.001	0.005 ± 0.001					
Androstenedione														
AM	Male	0.082 ± 0.023	0.062 ± 0.02	0.077 ± 0.015	0.073 ± 0.017	0.079 ± 0.035	0.102 ± 0.084	0.066 ± 0.014	0.084 ± 0.027	0.002	< 0.0001	0.6491075	0.93	0.2
	Female	0.079 ± 0.012	0.06 ± 0.024	0.072 ± 0.022	0.102 ± 0.05	0.083 ± 0.035	0.059 ± 0.013	0.057 ± 0.022	0.073 ± 0.023	F (6, 91) = 3.87	F (1, 91) = 282.39	F (1, 91) = 0.21	F (1, 7) = 0.009	F (6, 91) = 1.47
PM	Male	0.037 ± 0.011	0.024 ± 0.005	0.038 ± 0.013	0.027 ± 0.008	0.029 ± 0.008		0.028 ± 0.012	0.039 ± 0.01					
	Female	0.025 ± 0.004	0.022 ± 0.005	0.028 ± 0.006	0.029 ± 0.008	0.051 ± 0.032		0.021 ± 0.008	0.036 ± 0.005					
Androstenedione: Cortisol ratio														
AM	Male	0.026 ± 0.006	0.025 ± 0.013	0.024 ± 0.012	0.017 ± 0.003	0.016 ± 0.003	0.024 ± 0.011	0.023 ± 0.012	0.04 ± 0.006	< 0.0001	< 0.0001	0.90032394	0.71	0.024
	Female	0.027 ± 0.014	0.016 ± 0.002	0.029 ± 0.013	0.014 ± 0.006	0.015 ± 0.001	0.021 ± 0.002	0.039 ± 0.008	0.049 ± 0.019	F (6, 91) = 6.60	F (1, 91) = 111.85	F (1, 91) = 0.02	F (1, 7) = 0.14	F (6, 91) = 2.58
PM	Male	0.132 ± 0.045	0.074 ± 0.013	0.054 ± 0.043	0.041 ± 0.015	0.048 ± 0.023		0.05 ± 0.027	0.082 ± 0.042					
	Female	0.074 ± 0.026	0.069 ± 0.066	0.081 ± 0.054	0.059 ± 0.032	0.116 ± 0.134		0.037 ± 0.014	0.068 ± 0.023					
Testosterone: Cortisol ratio														
AM	Male	0.03 ± 0.009	0.032 ± 0.017	0.031 ± 0.021	0.019 ± 0.003	0.017 ± 0.004	0.024 ± 0.011	0.028 ± 0.014	0.054 ± 0.022	< 0.0001	< 0.0001	< 0.0001	< 0.0001	< 0.0001
	Female	0.004 ± 0.002	0.003 ± 0.003	0.004 ± 0.001	0.003 ± 0.003	0.002 ± 0.001	0.021 ± 0.002	0.011 ± 0.004	0.01 ± 0.001	F (6, 91) = 8.12	F (1, 91) = 74.91	F (1, 7) = 30.31	F (6, 91) = 6.47	F (1, 91) = 30.22
PM	Male	0.162 ± 0.063	0.099 ± 0.023	0.065 ± 0.058	0.051 ± 0.015	0.055 ± 0.034		0.063 ± 0.033	0.085 ± 0.043					
	Female	0.014 ± 0.003	0.014 ± 0.02	0.009 ± 0.006	0.01 ± 0.006	0.081 ± 0.128		0.01 ± 0.006	0.01 ± 0.006					

*awAUC* awakening AUC. P values refer to multi-way ANOVA using aligned rank transform comparing sex, time (i.e. date) and time of day.

**Fig. 2:**
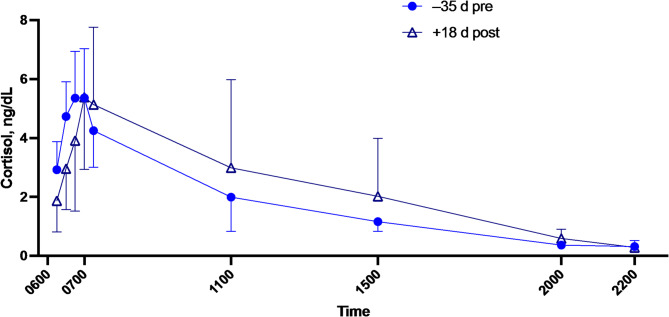
Salivary cortisol day curve 30 days before (filled circles) and 13 days after the expedition (triangles). Data are Mean ± SD. No significant difference was seen in pre and post area under the curve (multi-way ANOVA using aligned rank transform).

No significant change was seen in salivary day curve for cortisol, testosterone or androstenedione between 30 days before and 13 days after the expedition (pre- and post-expedition) 0.96 ± 0.46 versus 0.87 ± 0.73 ng.ml^-1^ hr, *p* = 0.9, (no sex difference or sex × time interaction was found; *p* = 0.8). (Fig. 2).

**Fig. 3:**
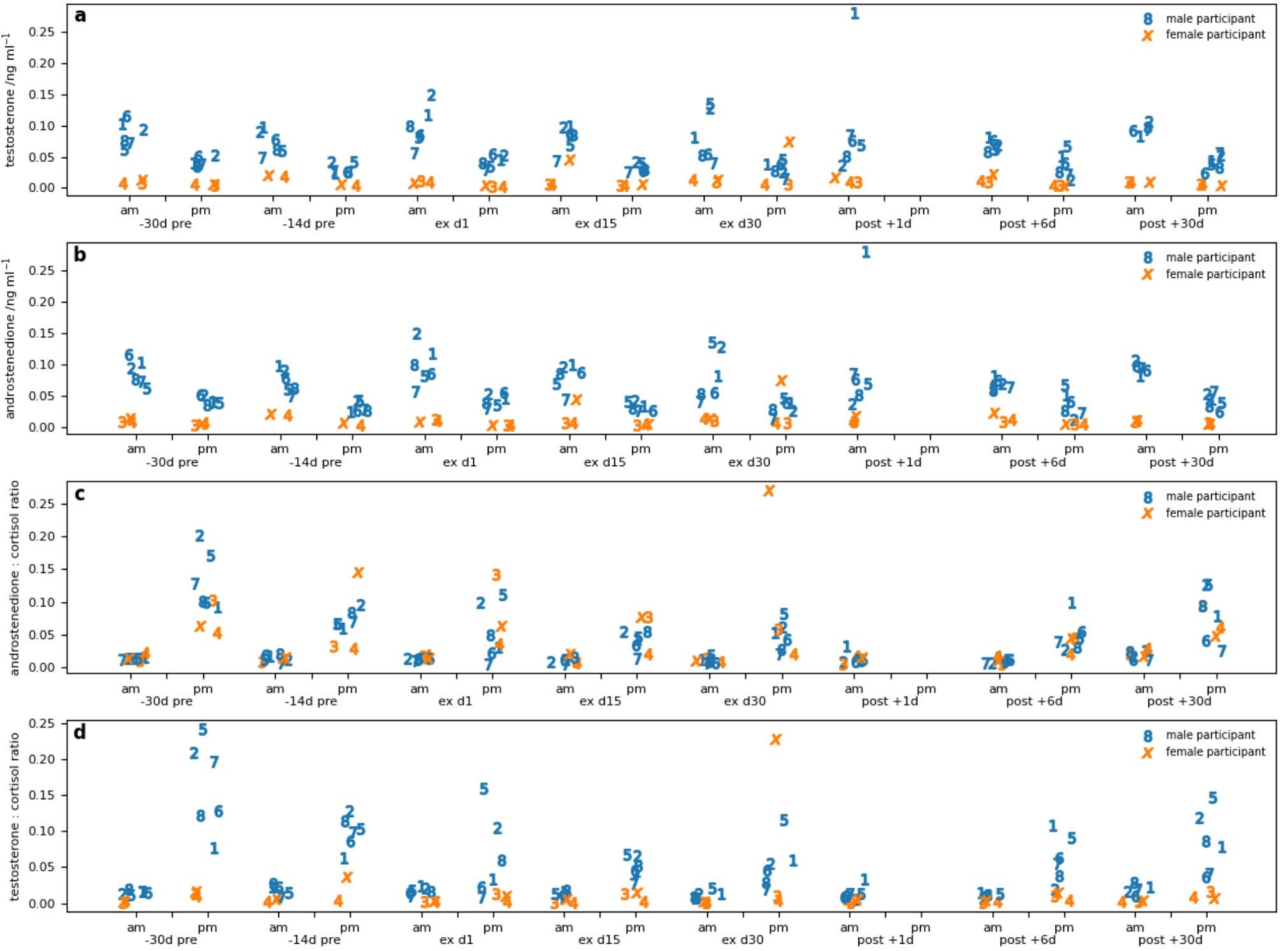
(**a**) Salivary testosterone. (**b**) Salivary androstenedione (A4). (**c**) Salivary androstenedione(A4): cortisol ratio. (**d**) Salivary testosterone: cortisol ratio.

Salivary testosterone concentrations were significantly higher among men than women, as expected (Fig. 3A). Morning salivary testosterone decreased during the expedition and recovered by Post + 1 and + 13 days with a greater effect seen among men than women (significant interaction of sex × time). Evening salivary testosterone increased relative to morning levels during the expedition and recovered afterwards (significant effect of time of day and interaction of time of day × time). Androstenedione concentrations were lower 14 days before the expedition than during, when they were stable. No sex difference in androstenedione was observed or interaction of sex × time (Fig. 3B).

The morning and evening ratios of androstenedione: cortisol and testosterone: cortisol demonstrated marked decreases during the expedition among men but were more stable among women and recovered to baseline after 13 days in all participants after the expedition (significant effect of time, and sex × time interaction; Fig. 3C and D).

**Fig. 4:**
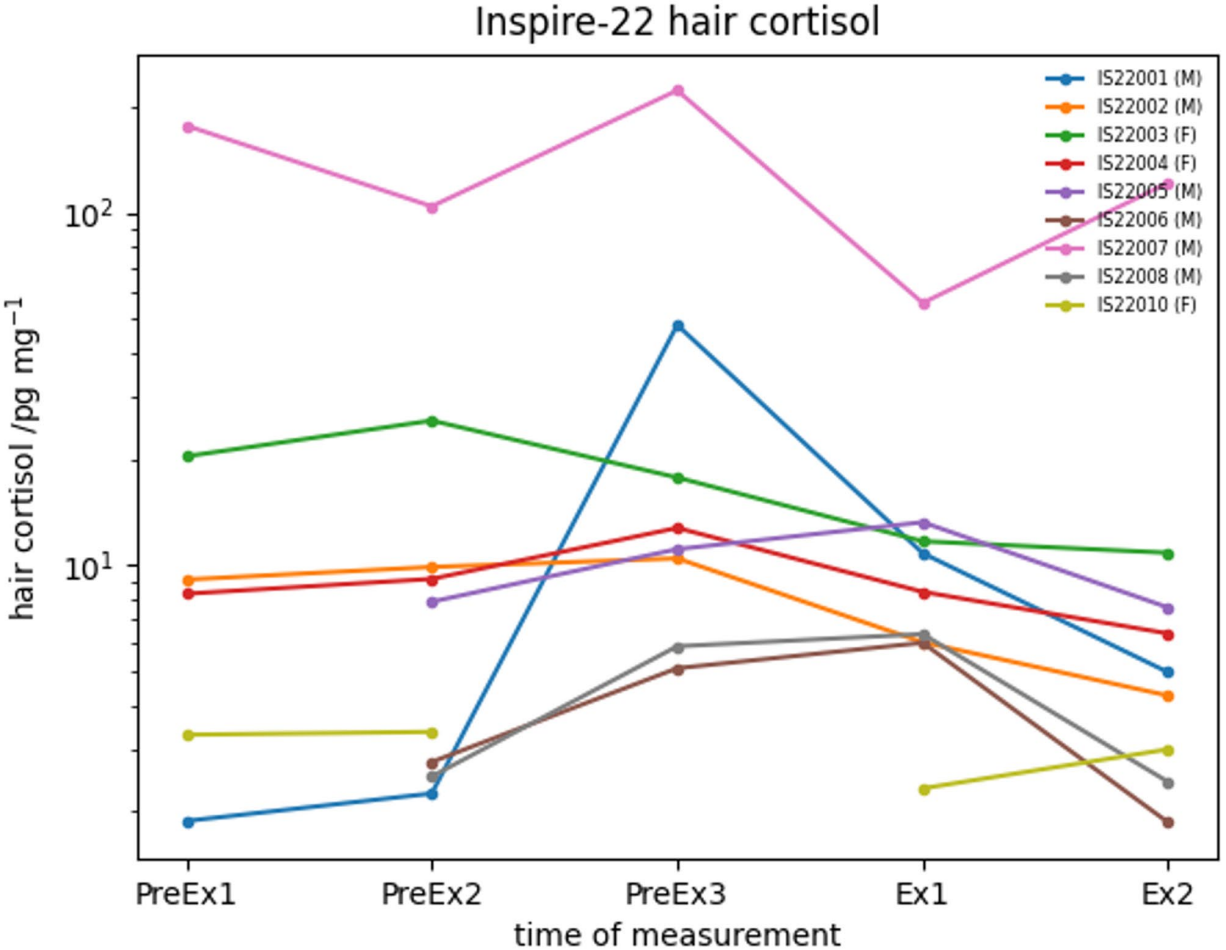
Hair cortisol concentration before and during the expedition. Left panel: all participants, Right panel: excluding IS2007. No temporal trend in hair cortisol was seen before or during the expedition (multi-way ANOVA using aligned rank transform).

Hair cortisol concentrations were markedly higher in one participant than the others (IS22007, male), which were not reflected in that participant’s saliva cortisol. Of the remaining 8 participants, 4 datapoints from 4 participants (3 male) were missing due to an insufficient hair mass. No temporal trend in cortisol hair levels was seen during the study (Fig. 4).

Plasma and serum hormones are shown in Table 2. No significant difference was seen in plasma cortisol over time or between sexes. Free testosterone concentrations were significantly higher among men throughout, as expected, and decreased during the expedition (significant effects of time, sex and sex × time interaction). An increase in free testosterone was observed in women but not men prior to the expedition, but levels after the expedition were no different to Pre – 30 days. Plasma androstenedione concentrations did not change over time, with no significant differences between men and women.

**Table 2:** Plasma and serum hormones before and after the expedition, standard units.

	Pre − 35 days	Pre − 14 days	Post + 6 days	Post + 13 days	*p* (time)	*p* (sex)	*p* (sex × time)
Cortisol, ng/mL							
Male	96.1 ± 21.5	86.4 ± 18.9	113 ± 22.3	101 ± 22.6	0.194	0.325	0.236
Female	168 ± 84.6	119 ± 75.2	137 ± 94	148 ± 47.8	F (3, 21) = 1.72	F (1, 7) = 1.12	F (3, 21) = 1.53
Calculated free testosterone, ng/dL							
Male	0.109 ± 0.017	0.109 ± 0.014	0.095 ± 0.026	0.106 ± 0.013	0.015	0.003	0.008
Female	0.002 ± 0.001	0.038 ± 0.02	0.002 ± 0.001	0.002 ± 0.001	F (3, 21) = 4.37	F (1, 7) = 20.704	F (3, 21) = 5.073
Androstenedione, ng/mL							
Male	0.448 ± 0.05	0.457 ± 0.129	0.521 ± 0.2	0.542 ± 0.186	0.864	0.061	0.405
Female	0.823 ± 0.121	0.604 ± 0.3	0.658 ± 0.322	0.698 ± 0.103	F (3, 21) = 0.25	F (1, 7) = 4.978	F (3, 21) = 1.017
FSH, mIU/mL							
Male	3.93 ± 1.66	4.45 ± 2.01	4.74 ± 2.01	4.08 ± 1.88	0.014	0.833	0.003
Female	2.95 ± 2.53	3.03 ± 2.9	4.59 ± 4.27	6.23 ± 4.06	F (3, 21) = 4.49	F (1, 7) = 0.048	F (3, 21) = 6.55
LH, mIU/mL							
Male	2.7 ± 1.29	3.37 ± 1.27	2.54 ± 0.987	2.74 ± 1.14	0.155	0.973	0.005
Female	1.69 ± 1.82	1.98 ± 1.79	2.97 ± 3.25	6.93 ± 8.39	F (3, 21) = 1.94	F (1, 7) = 0.001	F (3, 21) = 5.714
TSH, mIU/mL							
Male	1.14 ± 0.37	1.4 ± 0.6	2.26 ± 0.719	1.42 ± 0.454	< 0.0001	0.356	0.122
Female	0.971 ± 0.226	1.18 ± 0.247	1.41 ± 0.568	1.55 ± 1.09	F (3, 21) = 9.05	F (1, 7) = 0.978	F (3, 21) = 2.164
Free T4, ng/dL							
Male	1 ± 0.108	0.937 ± 0.059	0.945 ± 0.054	0.935 ± 0.074	0.089	0.875	0.773
Female	1.03 ± 0.11	0.89 ± 0.149	0.907 ± 0.118	0.903 ± 0.151	F (3, 21) = 2.48	F (1, 7) = 0.027	F (3, 21) = 0.374
Free T3, pg/mL							
Male	2.8 ± 0.213	2.81 ± 0.273	2.87 ± 0.278	2.74 ± 0.253	0.806	0.59	0.512
Female	3.04 ± 0.19	2.76 ± 0.378	2.75 ± 0.65	2.86 ± 0.602	F (3, 21) = 0.33	F (1, 7) = 0.318	F (3, 21) = 0.793
Total T3, ng/mL							
Male	0.95 ± 0.095	0.873 ± 0.086	0.98 ± 0.085	0.888 ± 0.12	0.268	0.661	0.237
Female	1.06 ± 0.1	0.873 ± 0.191	0.873 ± 0.237	0.917 ± 0.248	F (3, 21) = 1.41	F (1, 7) = 0.209	F (3, 21) = 1.527

*FSH* follicle-stimulating hormone, *LH* luteinising hormone, *TSH* thyroid stimulating hormone, *T4* thyroxine, *T3* triiodothyronine. P values refer to multi-way ANOVA comparing sex and time (i.e. date) using aligned rank transform.

Serum FSH and LH did not change during the study in men (Table 2). In women, both LH and FSH were suppressed prior to the expedition, and returned to or exceeded baseline concentrations after the expedition (significant sex × time interaction).

Thyroid stimulating hormone increased significantly after the expedition (Table 2). No changes were seen in any of free T4, free T3 or total T3 concentrations.

The correlation coefficients for blood and salivary androstenedione and testosterone were *r* = 0.76, *p* = 8.56 × 10^− 8^ and *r* = 0.09, *p* = 0.61, respectively. These are included in Supplementary Fig. 1.

## Discussion

INSPIRE-22 were the largest team ever to successfully complete an Antarctic traverse expedition and the first to conduct salivary steroid measures while doing so. Despite consuming a generous diet within nutritional guidelines^21^, participants sustained an average 7 kg weight loss, commensurate with previous expeditions^2,3,29^. Body mass loss comprised near linear loss of fat mass and highly variable changes in fat-free mass^21^. Weight loss was greater for male participants than females, even allowing for the comparatively small sample size. Despite the small sample size and exploratory nature of the study, in particular with respect to sex differences, we are able to make a number of qualified observations.

Contrary to our hypothesis, morning cortisol increased during the expedition (both a greater peak and AUC after awakening). Some participants demonstrated a modest increase in evening cortisol but overall, the diurnal slope was increased. We had hypothesised that 24-hour daylight would be associated with reduced diurnal slope, due to the effect of photic stimulus on the suprachiasmatic nucleus resulting in hypothalamic dysregulation^20^. Increased diurnal cortisol variation with 24-h sunlight is, we believe, a novel finding and contrasts with those from a less physically demanding expedition, where loss of the diurnal variation was noted^17^. We surmise that the physical demands of the expedition and the fixed daily schedule eclipsed the effect of 24-hour sunlight. Over the 30 days following the expedition, diurnal cortisol variation returned to baseline.

Hair cortisol measures were stable throughout the expedition and beforehand. By contrast, in a study of 6 women skiing 1,700 km across Antarctica, average hair cortisol increased dramatically during the expedition compared with beforehand, perhaps suggesting greater metabolic demands and/or psychological stress than INSPIRE22^30^. Similarly, in that study, one participant demonstrated markedly higher hair cortisol levels during the expedition which were not reflected in morning or evening salivary levels before or afterwards. The present study found that even during the expedition, morning and evening cortisol levels did not reflect the average cortisol concentration measured in hair in these outliers. Hair cortisol is a long-term marker of HPA axis activity which is influenced by a wide array of sociodemographic, situational, environmental and genetic factors^31^. Higher hair cortisol may be associated with lower cortisol stress reactivity^32^. The marked inter-individual variations seen in hair cortisol suggest that some individuals face far greater HPA axis adaptation in response to a given stressor.

Sex comparisons are qualified because of the small cohort. The diurnal cortisol profiles and cortisol response to wakening did not differ between men and women during the expedition. However, taken together, all cortisol concentrations suggested a slight increase among women during the expedition compared with men. This difference was not reflected in hair cortisol. Women are well adapted to survive an energy deficit, through greater storage and utilisation of fat mass than men^33^, and preservation of lean and fat tissue mass as we observed during INSPIRE22^21^. No major sex differences in HPA axis activation were seen in this small sample, suggesting that other hormonal factors such as oestrogen impact lipid utilisation^34^.

Plasma cortisol did not change after the expedition or appear to differ between sexes. However, blood was sampled several days after the expedition, during which time recovery of steroid levels would be expected^35^. Moreover, the stress of venipuncture is itself associated with elevated cortisol response, which tends to habituate with repeated testing^30,36^.

The decrease in morning salivary testosterone concentrations we observed appeared more marked among men than women and our data suggested recovery over the following 30 days. Morning plasma testosterone remained low 6 days after the expedition, before recovering to baseline by the end of the study. This contrasts with the study of Woods et al., which demonstrated an increase in testosterone after a shorter ski traverse in 19 men, during which participants conserved body mass and percentage body fat^1^. When sustained physical training is associated with energy deficit and weight loss, male testosterone levels usually fall, as demonstrated previously over similar^37,38^ or shorter durations^39,40^. It follows that body mass loss and a catabolic state are likely to explain the decreased testosterone we observed. In a less arduous study of camping on Antarctica, Moraes et al. demonstrated preserved testosterone levels but loss of normal diurnal variation^17^. As with cortisol, the preserved diurnal variation we observed for testosterone could be explained by the physical activity routine.

Comparison of testosterone levels between men and women must accommodate vastly different and non-overlapping reference ranges^18^. Reliance on testosterone to compare sex differences in anabolic adaptation will increase the risk of type 2 error, hence we avoided this by using an LC-MS/MS methodology to measure androstenedione, a weak androgen produced by the adrenals in similar concentrations in men and women. Plasma and salivary androstenedione followed similar visual trends to testosterone (outliers consistently demonstrated high or low concentrations in both androgens), however in absolute terms, androstenedione levels were similar between sexes. Unlike testosterone, a significant interaction of sex with time was not seen for androstenedione in this small sample. Consistent with our hypothesis, higher androstenedione: cortisol ratio was demonstrated among women than men, driven by increased cortisol in two participants, rather than a sex difference in androstenedione variance. It is unclear whether the discrepancy between testosterone and androstenedione was because a change in testosterone is more likely to be detected in men, who have far higher levels, or because men are more susceptible to the endocrine consequences of a catabolic state. Both appear likely^35^. Our findings were comparable to adaptations seen in overtraining syndrome^41^. Controlled studies are required comparing adaptive responses to exercise and energy deficit between the sexes.

Basal LH and FSH suggested a degree of central HPG axis suppression in women after the expedition, but not men, which returned increased within 13 days. Our findings are consistent with a study of 6 women who crossed Antarctica, where pituitary gonadotroph function was suppressed beforehand, but preserved during the expedition despite marked HPA axis activation, and returned to normal levels 2 weeks later^3^.

The perturbations in serum thyroid hormones we observed were consistent with the ‘polar T3 syndrome’, reported in men and women during exposure to a polar environment, reflecting increased uptake of T3 to maintain basal metabolic rate and promote thermogenesis^13,17^. In warmer climates (greater than 0 ℃) less adaptive thermogenesis is expected, leading to mild upregulation of TSH without increased tissue T3 uptake, and occasionally increased circulating T3 levels^13^. Our observations imply central activation of the HPT axis but the intensity and/ or duration of cold exposure was insufficient for peripheral tissue adaptation to cause low T3 concentrations, as seen in classical polar T3 syndrome^13^. Similar thyroid hormone changes were seen in women undertaking an Antarctic crossing^3^, in women during stressful military training^42^, and during camping in Antarctica after sailing^17^. Woods et al.^1^demonstrated increased TSH *and* free T3 levels after a shorter ski expedition, suggesting a milder manifestation. Central activation of the HPT axis serves to increase basal metabolism and non-shivering thermogenesis.

Strengths of our study include the inclusion of a mixed sex cohort and the use of detailed frequent sample collection during the expedition, which to our knowledge have never previously been attempted in such an austere, remote setting. The use of saliva sampling allows point measures of steroid hormones during a dynamic activity, without sampling itself causing pain or stress. These samples were complemented with contemporaneous morning blood sampling to allow comparison of serum and saliva steroid hormone concentrations with published studies normally relying on blood steroid concentrations. The use of LC-MS/MS, as a gold standard technique for steroid hormone measurement, allowed simultaneous and precise measurement of steroid hormones across a wide analytical range, ensuring isobaric (same mass) compounds were separated^23^. Our study has several limitations. While this was the largest ever expedition of its kind, it was nevertheless exploratory the findings should be interpreted cautiously in the context of the small cohort particularly of women participants and therefore consideration of the impact of hormonal contraception, menstrual cycle stage and pre/postmenopausal status of women, on salivary steroid hormone concentrations must be treated with extreme caution. Interpreting the adaptability of steroid hormones to such an extreme environment is limited the complex signalling networks which exist, with multiple input points, parallel signalling pathways, feedback and feedforward mechanisms etc. The observational nature of the study meant it was not possible to delineate individual stressors to which participants were exposed (e.g. 24 h-daylight, exercise, energy deficit). Therefore, our results are primarily descriptive, and conclusions must be interpreted with caution.

In conclusion, during an arduous 51-day Antarctic expedition, saliva sampling from 9 participants demonstrated morning activation of the HPA axis, likely highlighting a greater effect of exercise on HPA axis variation than 24-hour daylight. Testosterone levels were suppressed in men and no change was seen in androstenedione in male or female subjects. Diurnal variations in glucocorticoids and androgens were preserved overall. There was little evidence to suggest a sex dimorphism in HPA axis activity during the expedition, with no difference in circadian patterns. Gonadotrophins were modestly suppressed, thyroid stimulating hormone increased, while thyroid hormones were stable, consistent with descriptions of polar T3 syndrome. These findings suggest a temporary general adaptation to prolonged and arduous exercise in an austere environment mediated by changes in anterior pituitary function.

## Electronic supplementary material

Below is the link to the electronic supplementary material.

Supplementary Material 1

## Data Availability

The datasets used and/or analysed during the current study available from the corresponding author on reasonable request.
