# Supplementary material for: Preserved circadian variation in cortisol and androgens during a ski traverse of Antarctica in summer
Source: Sci Rep. 2025 May 22;15:17726. doi: 10.1038/s41598-025-01165-1 (PMC12095593; doi:10.1038/s41598-025-01165-1)
Supplement: Supplementary file 1 — Supplementary Material 1 [file 41598_2025_1165_MOESM1_ESM.docx]

**Supplementary figure 1: comparison of salivary and blood androgens**


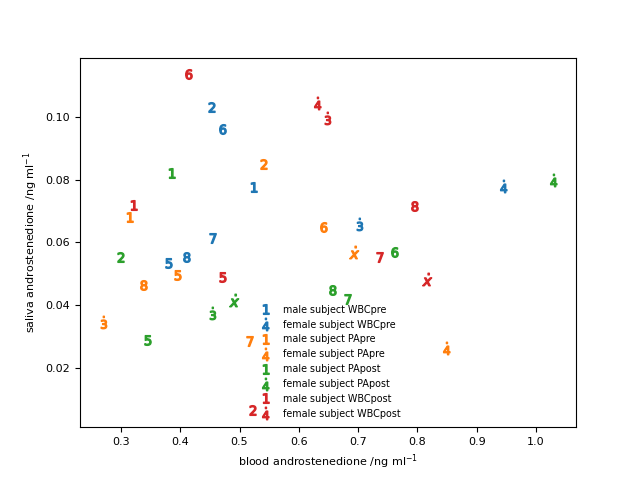

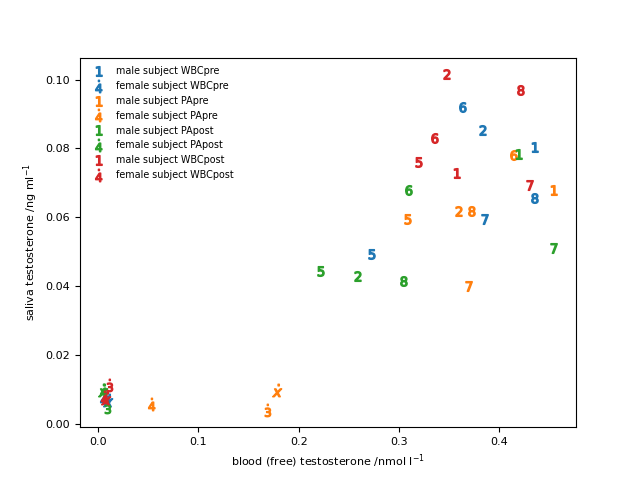


Spearman’s rank correlation coefficients for blood and salivary androstenedione and testosterone were r = 0.76, p = 8.56 x 10^-8^ and r = 0.09, p = 0.61, respectively. WBC: whole body calorimeter (the location for visits 30 days pre and 30 days post expedition); PA: Punta Arenas (the location for visits 14 days pre and 6 days post expedition).
